# Hybrid HCNN-KNN Model Enhances Age Estimation Accuracy in Orthopantomography

**DOI:** 10.3389/fpubh.2022.879418

**Published:** 2022-05-30

**Authors:** Fatemeh Sharifonnasabi, Noor Zaman Jhanjhi, Jacob John, Peyman Obeidy, Shahab S. Band, Hamid Alinejad-Rokny, Mohammed Baz

**Affiliations:** ^1^Department of Computer Science & Engineering, School of Computing & IT (SoCIT), Taylor's University, Subang Jaya, Malaysia; ^2^Department of Restorative Dentistry, Faculty of Dentistry, University of Malaya, Kuala Lumpur, Malaysia; ^3^Charles Perkins Centre, Faculty of Medicine and Health, University of Sydney, Darlington, NSW, Australia; ^4^Future Technology Research Centre, College of Future, National Yunlin University of Science and Technology, Yunlin, Taiwan; ^5^BioMedical Machine Learning Lab (BML), The Graduate School of Biomedical Engineering, University of New South Wales (UNSW) Sydney, Kensington, NSW, Australia; ^6^UNSW Data Science Hub, The University of New South Wales, UNSW Sydney, Kensington, NSW, Australia; ^7^Health Data Analytics Program, AI-enabled Processes (AIP) Research Centre, Macquarie University, Macquarie Park, NSW, Australia; ^8^Department of Computer Engineering, College of Computer and Information Technology, Taif University, Taif, Saudi Arabia

**Keywords:** dental age, estimation, Orthopantomogram, convolutional neural network, k-nearest neighbor, Biomedical machine learning

## Abstract

Age estimation in dental radiographs Orthopantomography (OPG) is a medical imaging technique that physicians and pathologists utilize for disease identification and legal matters. For example, for estimating post-mortem interval, detecting child abuse, drug trafficking, and identifying an unknown body. Recent development in automated image processing models improved the age estimation's limited precision to an approximate range of +/- 1 year. While this estimation is often accepted as accurate measurement, age estimation should be as precise as possible in most serious matters, such as homicide. Current age estimation techniques are highly dependent on manual and time-consuming image processing. Age estimation is often a time-sensitive matter in which the image processing time is vital. Recent development in Machine learning-based data processing methods has decreased the imaging time processing; however, the accuracy of these techniques remains to be further improved. We proposed an ensemble method of image classifiers to enhance the accuracy of age estimation using OPGs from 1 year to a couple of months (1-3-6). This hybrid model is based on convolutional neural networks (CNN) and K nearest neighbors (KNN). The hybrid (HCNN-KNN) model was used to investigate 1,922 panoramic dental radiographs of patients aged 15 to 23. These OPGs were obtained from the various teaching institutes and private dental clinics in Malaysia. To minimize the chance of overfitting in our model, we used the principal component analysis (PCA) algorithm and eliminated the features with high correlation. To further enhance the performance of our hybrid model, we performed systematic image pre-processing. We applied a series of classifications to train our model. We have successfully demonstrated that combining these innovative approaches has improved the classification and segmentation and thus the age-estimation outcome of the model. Our findings suggest that our innovative model, for the first time, to the best of our knowledge, successfully estimated the age in classified studies of 1 year old, 6 months, 3 months and 1-month-old cases with accuracies of 99.98, 99.96, 99.87, and 98.78 respectively.

## Introduction

Age estimation in living and deceased individuals has always been important in pediatric studies, pathological complications, and forensic medicine (1, 2). Human bone age can be estimated from foot, shoulder, ankle, hip, elbow, cervix, ankle, and teeth bones (3–5). The accuracy of the calculated age from a bone specimen depends on genetic and environmental factors such as age, race, smoking, lifestyle, famine and natural disasters (6). While current age estimation methods, for example, Greulich-Pyle or Tanner–Whitehouse method, can accurately estimate age from radiological examination of the left hand in children, age assassination complexities significantly increase when comparing young adults to adolescents. The absence of formal age documentation makes the process even more complicated despite recent discoveries in anthropometric fields demonstrating the importance of other bones, such as the pelvis, in determining bone age (3, 4). OPG or dental radiographs remained to be the primary forensic concern (7).

Orthopantomography, also known as an OPG or dental radiographs, provides a wide panoramic view of the lower face bones (8). OPG displays all the teeth on both jaws. OPG also depicts the jawbone and the temporomandibular joint (TMJ) on the same radiography film. The dental OPG images have been suggested to be more effective when compared to other bones of the body X-ray images. Teeth bones are long-lasting and resistant to high temperatures (2) and organisms which decompose a dead body (9). The Malaysian Institute of Forensic Medicine claimed that the X-ray images of teeth are the primary and the most used method in estimating age (10, 11). However, the traditional image processing approach for OPG age estimation is still relatively expensive, laborious and requires long-term monitoring, increasing potential radiation exposure (12).

Traditional image processing for dental age measurement is a manual method that may include several steps such as segmentation, feature extraction, image pre-processing, classification, or regression. Each of these steps is error-prone and can induce variations in the outcome. For example, in an identical age range, the dry bone image will be different from the wet bone in radiography scan. In recent years, Machine Learning/Deep Learning techniques have been widely used to identify patterns in complex data such as clinical imaging, genomics, bioimaging, and phenotypic data (13–17). Machine learning algorithms for example, NN, SVM, KNN, Decision Tree, displayed promising ability in prediction and classification (18–28) including estimation of bone age (29, 30). The medical and biological datasets are increasing rapidly. To analyse such big and complex data, artificial intelligence and machine learning algorithms become most popular (31–41). Therefore, it is important to implement novel techniques to uncover the medical and biological patterns. In particular machine learning and deep learning techniques have been widely used to analyse imaging data (23, 37, 42).

Deep learning-based methods, also known as end-to-end learning-based methods, such as convolutional neural networks, are either unsupervised that operate directly on the input images and generate the desired output without intermediate steps such as segmentation and extraction or supervised. Supervised learning (SL) models utilize a learning function that requires example input-output pairs for the model validation. The supervised approach runs the comparison between a radiographic image of each subject to an existing reference (i.e., labeled images), including gender and age information (43). Nevertheless, most of these methods require a large amount of labeled data.

Collecting and labeling large datasets are usually expensive, time-consuming, or sometimes impossible. Thus, in recent years, Semi-Supervised Learning (SSL) has emerged. Leveraging both labeled and unlabelled data, SSL has been proved to be a practical approach. Transfer learning (TL), an SSL-based model) aiming to utilize training knowledge gained from one data set to analyse another using a few-shot classification framework (44, 45). Moreover, unprocessed image databases, which usually can be fed to conventional ML or DL models, are prone to inaccurate class recognition. Extracting specific characteristics that accurately represent behavior in different environmental conditions is also a time-consuming task. (11, 46). Hence, choosing a classifier that can differentiate behaviors in terms of diversity within and outside each class is one of the challenges in recognizing behavior. Therefore, converting raw data into attribute vectors systematically and using effective attribute extractors using an engineering approach is required for proper classification (47–49).

Despite these improvements developing and training deep neural networks remained challenging and time-consuming. Thus, Transfer learning (TL) can use the pre-trained deep network to perform data classification, attracting increasing attention (50, 51) this article proposed an innovative automated machine learning model approach to estimate bone age. This approach is based on a deep convolutional neural network (52). We improved the accuracy of age determination in dental panoramic images with approximately 6-month intervals. The proposed method determines the precise age in the range of 15 to 23 years old that is divided into nine age groups. Each age group includes subcategories of images with 1 year, six, three and 1-month(s) intervals. This method combined the methodology of features extraction of the convolutional neural networks with the nearest neighbor feature to analyse and classify information and features in dental images.

**Figure 1:**
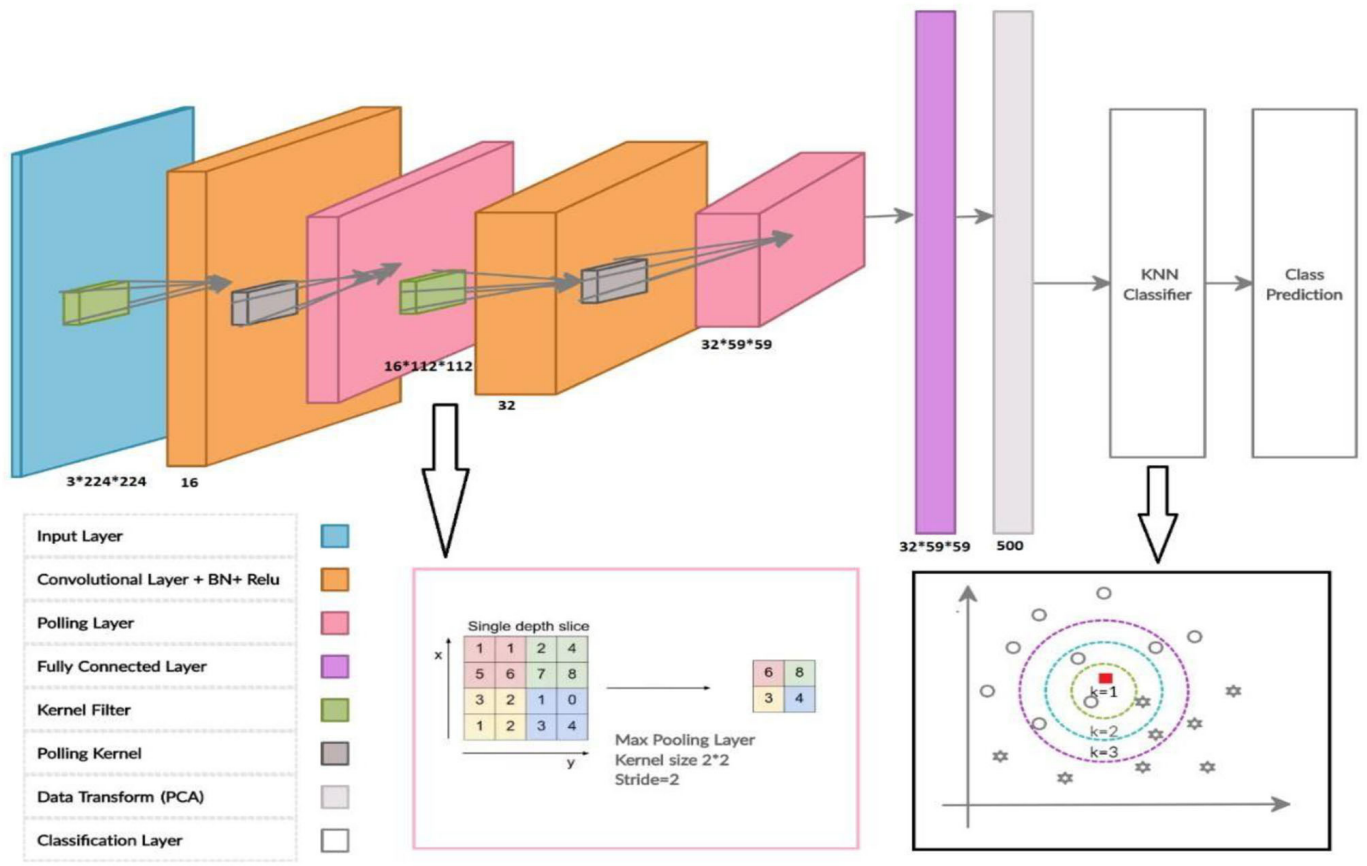
The architecture of the proposed HCNN-KNN model.

More specifically, the architecture of the proposed HCNN-KNN model used in this study consists of four steps (Figure 1). Initially, a convolutional neural network (CNN) model was trained on OPG images. Then, a fully connected layer generated from CNN was fed into a principal component analysis (PCA). The PCA performed data transformation and dimension reduction. In the last step, data were classified using the KNN algorithm. The details of the prediction process of the classes in the data set were explained in the following sections.

### Related Literature

Age estimation based on dental X-ray images remained a critical field to develop. Tooth bones are immortal and can last more than 20 years through post-mortem decomposition. This made the tooth an essential part of the forensic investigation (53). Although the number of bone age estimation models has increased, most remain in the research state with slow or no transition in the industry. Computerized methods for bone age estimation based on OPG images are currently limited to ± 6 months precision (52). Moreover, current Deep neural network algorithms used to estimate the age using dental X-ray images are, for example, AlexNet and ResNet. Training these algorithms requires large imaging datasets. For instance, Houssein et al. used a dataset with 1,429 dental X-ray images to reach acceptable output parameters (11).

In another study, Avuçlu et al. (54) used 1,315 dental images and 162 different dental classes to morphologically predict the age and gender in dental X-ray images with 95% accuracy (54, 55). Farhadian et al. (30) applied Transfer learning to reduce using a neural network that relied on dental data to alter age assessment. The age group of between 14 and 60 was used, and the overall data sample size for the research was 300 images. With an MAE of 4.12 years, the neural network methodology showed a reasonably accurate outcome (30). The neural network errors were smaller than the regression model's estimation errors, with the test data set being RMSE of 10.26 years and MAE of 8.17 years.

Alkaabi et al. (8) examined different Convolutional Neural Networks s AlexNet, VGGNet, and ResNet. Architectures for age estimation. They used common CNN architectures to perform forensic dentistry's automatic age calculation without any modifications. Using Capsule-Net, predicting age estimation from dental images was also performed, which depicted 36% higher accuracy than the CNNs model. The Capsule-Net model based on transfer learning reached a cumulative accuracy of 76% for ± 1-Year-old sample OPG images.

Tao et al. (29) has introduced the Multilayer Perceptron Neural network to estimate dental age. The experiments are carried out on a dataset composed of 1,636 samples. It was also experimentally confirmed that this latest feature set makes the dental age estimation more reliable for sample OPG images of ± 1-Year-old individuals' sample OPG images.

Kim et al. (56) applied a deep learning algorithm, based on CNN neural, to X-ray images of 9,435 cases (4,963 male, 4,472 female). Data in this cohort was sorted into three-age gatherings. Their study suggests that deep learning algorithms based on CNN neural networks show that the proposed approach functions evaluated based on a database of panoramic dental radiographs and worked well for accuracy.

Banar et al. (57) also implemented a fully automatic method leveraging the total capacity of the deep learning approach on their study of a dataset of 400 OPGs x-ray images with 20 OPGs per category and per gender. To overcome the limitations of having a dataset with a limited number of embodiments, the Barnar group employed a transfer learning approach using pre-trained CNNs, and data augmentation. Their study significantly reduced the imaging assessment time over the current conventional method. For example, they have reported that the entire automatic workflow took 2.72 s to compute on average. Given the small size of the dataset, this pilot study indicated the strength of transform learning and suggested a completely automated solution capable of demonstrating outcomes not inferior to manual assessing the images.

Tuan et al. (58) introduced a semi-supervised fuzzy clustering algorithm for pre-image processing and segmentation of dental X-ray images. Their study showed that their recommended work has superior accuracy than the initial semi-supervised fuzzy clustering and several related approaches.

In the Department of Dentistry and Study of University Sains Islam Malaysia, an age evaluation approach was tested on Malaysian adolescents between the ages of 1 to 17. Initially, the first to the third teeth were segmented, and the invariant deformation characteristics were then collected based on a deep learning method. The designed DCNN model was used to extract a broad range of features in the hierarchical layers, for example, invariance of size, rotation and deformation for sample OPG images of ± 1-Year-old individuals.

## Materials and Methods

### Image Pre-processing and Data Augmentation

Prior to utilizing images in our model, we performed image processing, adjusted the contrast and highlighted the images, removed extra margins (cropping), and normalized the image pixel values at edges from zero to one. In this study, data augmentation was performed by mirroring and data duplication with rotation.

Data were classified before, Splitting for Training the model. To perform the classification, the data in the dataset were divided into two categories: train data and validation data. The cross-validation was performed with 50–50, 70–30, and 20–80 train and validation split. To take an example, 20–80 split the 20% of the data for validation and 80% for training the model. Obtaining acceptable results on various test data will indicate the correct performance of the proposed model. Thus, to further enhance the validation of the model, we perform additional validation of the trained model using a data set that the model was seeing for the first time. The additional validation dataset included various features such as OPGs of different races, with different genders and at different ages.

### The Architecture of HCNN-KKN Proposed Model

**Figure 2:**
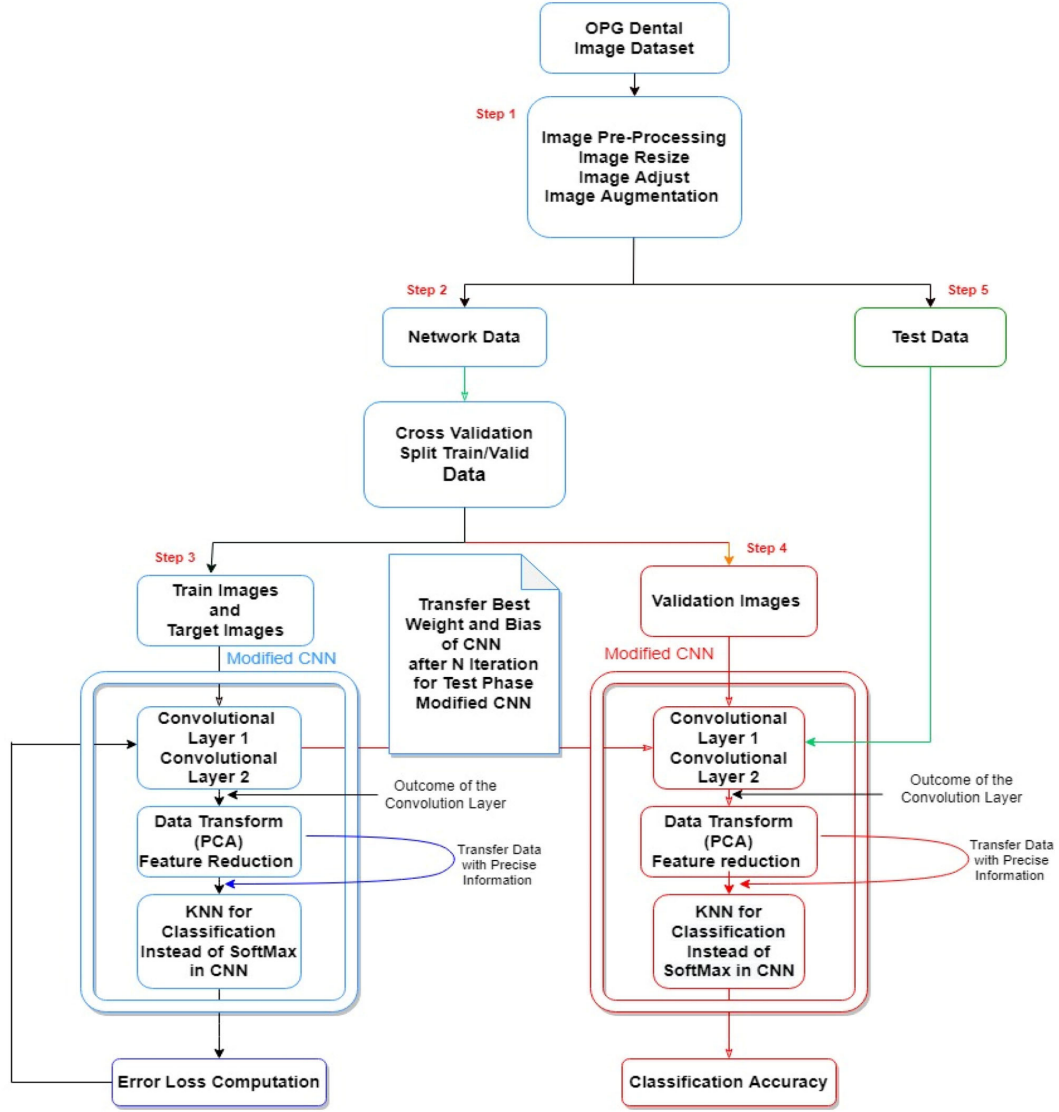
The conceptual framework for our hybrid model, dental image processing and classification processes.

In this study, we proposed a model based on a combination of the HCNN-KKN. Steps in our hybrid model depicted in Figure 2 for pre-image processing and the dental classification are as follows:

The architecture of the proposed HCNN-KNN model included an inputs layer, two Convolutional layers, a fully connected layer, a PCA transform data layer, and a KNN layer for classification (Figure 1). Using RGB images (224 x 224 pixels) as input, each image was fed to a convolutional layer (i.e., the second layer) containing 16 convolutional filters and a kernel size of 5 and 2 paddings. Low-level features like edges, blobs, shapes were obtained. In addition to the 16 convolutional normalization filters, batch normalization (16 layers), followed by a non-linear activation function via rectified linear unit (ReLU) were performed. The convolutional layer is termed “conv1”. Maxpooling layer downsampled the model with stride 2 and 2 paddings.

As a result, the Maxpooling layer reduced the dimensions of the images to 112 ×112 pixels, and the final output of this layer is equal to 112 ×112 ×16. These images were fed to the second convolutional block with a similar structure to the first. The later conventional block has 32 filters with 5 and 2 paddings kernel, resulting in the 56 ×56 ×32 images output. In the following step, all the fully connected layers were used to connect all the neurons in different layers (Table 1). To reduce the less important features to the next layer in the CNN model, the layers are connected with a 50% dropout between fully connected layers. We then applied a classification approach on a cohort of individuals between 15 to 23 years old and stratified all the cases with intervals of ± 6-months, ± 3-months, and ± 1-month. This resulted in 9 categories for each year (+_ Year), 18 categories for the 6 months intervals (each 6 months), 36 categories for each trimester of each year (each 3 months), 108 for every 12 months from each year (each 1 month). This resulted in the last fully connected layer to modify nine classification tasks with parameters like bias learn rate factor and weight learn rate factor. Figure 1: Illustration of the CNN Network elements.

**Table 1:** A display of the parameters used in the CNN network.

	**Type**	**Filter size**	**Stride**	**# Filters**	**FC**	**Input**
Layer1	Convolution	5 ×5	2 ×2	16	-	224 ×224 ×3
Layer2	Maxpooling	2 ×2	2 ×2	-	-	224 ×224 ×16
Layer3	Convolution	5 ×5	2 ×2	32	-	112 ×112 ×16
Layer4	Maxpooling	2 ×2	2 ×2	-	-	112 ×112 ×32
Layer5	Fully Connected	-	-	-	*	59 ×59 ×32

*9 for Dataset on Year, 18 for a dataset on ± 6-month, 36 for a dataset on ± 3 month and 108 for a dataset on ± 1 month.

In this step, a combined CNN model with KNN is used as a classifier to evaluate the class event instead of the SoftMax probability layer. Thus, before the data enters a fully connected layer of KNN layers, we proposed that in this model, the data were transferred in the space with more precise information and smaller dimensions using the principal component analysis method so-called “Fully_PCA”. Therefore, fully connected layer neurons after PCA data transformation and reductions are considered overlays of the KNN classification layer. In this case, the KNN input is assumed to be 500.

The HCNN-KNN model algorithm pseudo-code proposed in this bone age study is based on dental images. In the KNN network, the distance metric used is the Euclidean Distance metric and the “Ks” are 1, 2, 3. The model is trained using an SGD momentum optimiser with a 0.0001 learning rate. The model is then trained for 10 epochs with a mini-batch size of 16 images. After learning the network and classification operations, the error loss rate is obtained to evaluate the proposed network model in subsequent epochs of network learning.

### Evaluating the Proposed Model on Validation Data

The network neurons' optimal weight and bias were evaluated following the network training step. The validation data, segmented according to Figure 2 (step 2), was then categorized on the CNN network with optimal parameters. The components of the classification algorithms were determined according to the training phase process and the criteria that allow the data to pass through the CNN-PCA-KNN layers. Finally, the identification rate was evaluated in which accuracy of the index was selected according to the Confusion Metrics. In brief, the sum of TP + TN (TP: the number of times the class is upbeat and correctly detected, FP: the number of times the class was positive and undiagnosed), divided with the sum of TP + FP + TN + FN. TP is the number of times the class is positive and correctly detected, FP abbreviation for the number of times the class was positive and undiagnosed. The TN, denotes the number of times the class is incorrect (negative) and correctly diagnosed. The FN represents the number of times each class is erroneous (negative) or not correctly recognized.

### Validating the Proposed Model on a Test Dataset

To further validate the accuracy of the proposed model, we used test data which the model had not seen before, and was not part of the training dataset. This dataset was collected separately from the trained dataset and had different properties. This data combines X-ray OPG images of other races, with different genders and at different ages. This data enters the CNN network as a validation step and is categorized after passing through conv1, pooling1, conv2, pooling2, fully connected layer, PCA layer, and KNN layer. A vital feature of this step is the validation of the proposed model. Achieving acceptable results on various test data will indicate the satisfactory performance of the proposed model.

### Computational Hardware Requirement

For this study, we used an NVIDIA GeForce GTX 1080 8GB, 8GB memory, Intel Core i7, and 3.40GHz. We also used Python libraries NumPy, Matplotlib, Sklearn, Metrics, PyTorch, Torchvision to perform the analyses.

## Result and Discussion

We discussed the database used in this article, the tools, and the architecture introduced in the following. Finally, we evaluated our proposed model on the dataset.

### Datasets Description

Dental OPG is a panoramic radiograph that scans the upper and lower jaws with a two-dimensional view that shows a semicircle from ear to ear. The used images in this study are dental OPG data, collected from dental teaching institutes and private dental clinics in Malaysia. These collected data with the different image sizes are resized before importing into the proposed model. The original image size for all data has been changed to 600 ×1,024 pixels.

**Figure 3:**
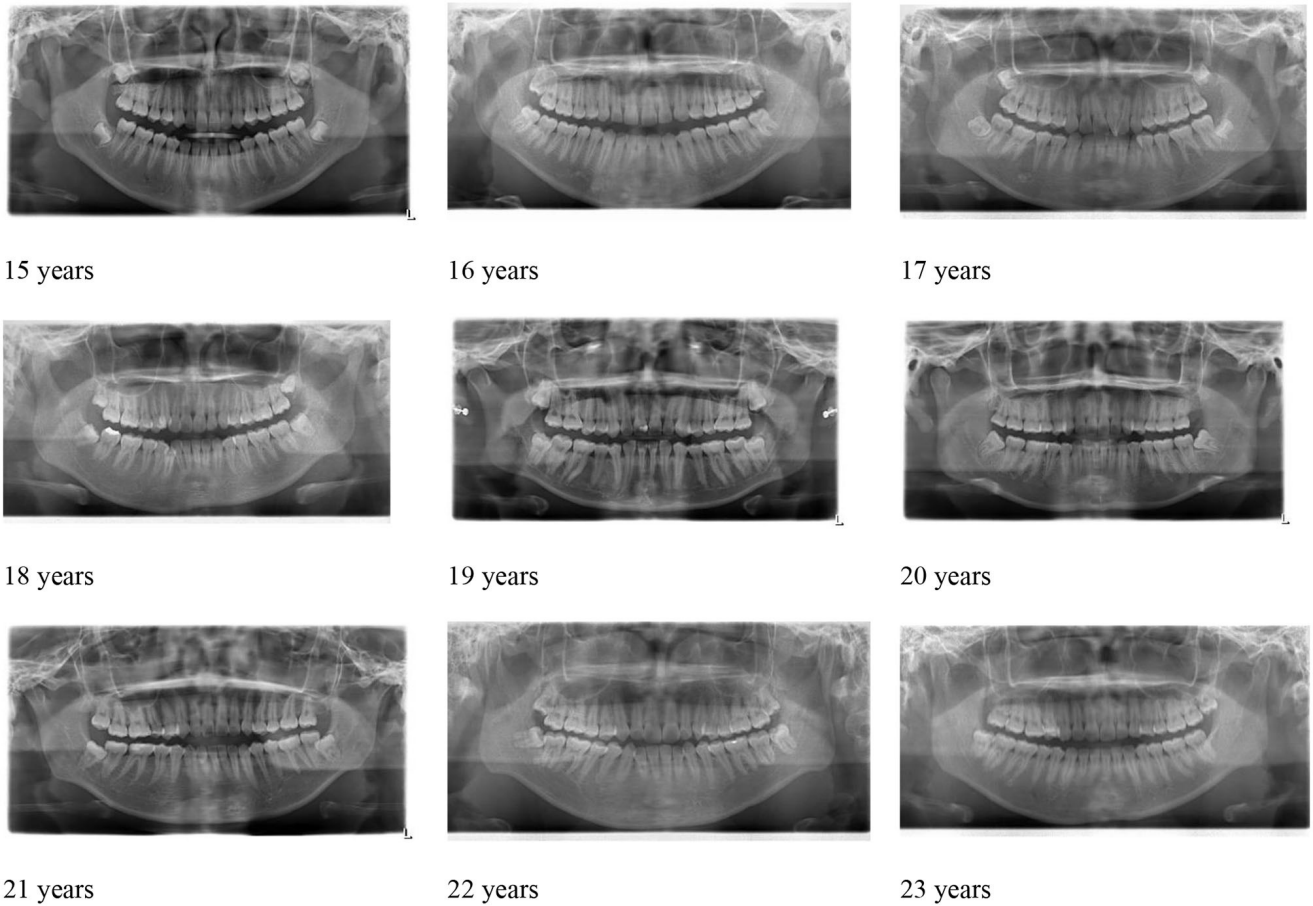
Representation of each age class.

Subjects from this study were randomly selected from 1,922 patients between 15 and 23 years of age, and this age range determines the age of minors which could help the model find more specific features and train the model more accurately, particularly for smaller time intervals. Moreover, to further validate the model, we used a dataset that hasn't been presented to the model during conventional training or the initial validation. Using an additional dataset including 130 random images, we showed that pre-training the model on such a prominent age recognition features (i.e. shorter time intervals, form of age range by year, age range by month, by season (3 months), and 6 months (Dataset on Year, on ± 6 months, on ± 3 months, and ± 1 month), allows us to evaluate our proposed method on a test database to enhance the performance of the proposed method (further details are provided in the experimental Result, Table 1, Figure 3).

**Figure 4:**
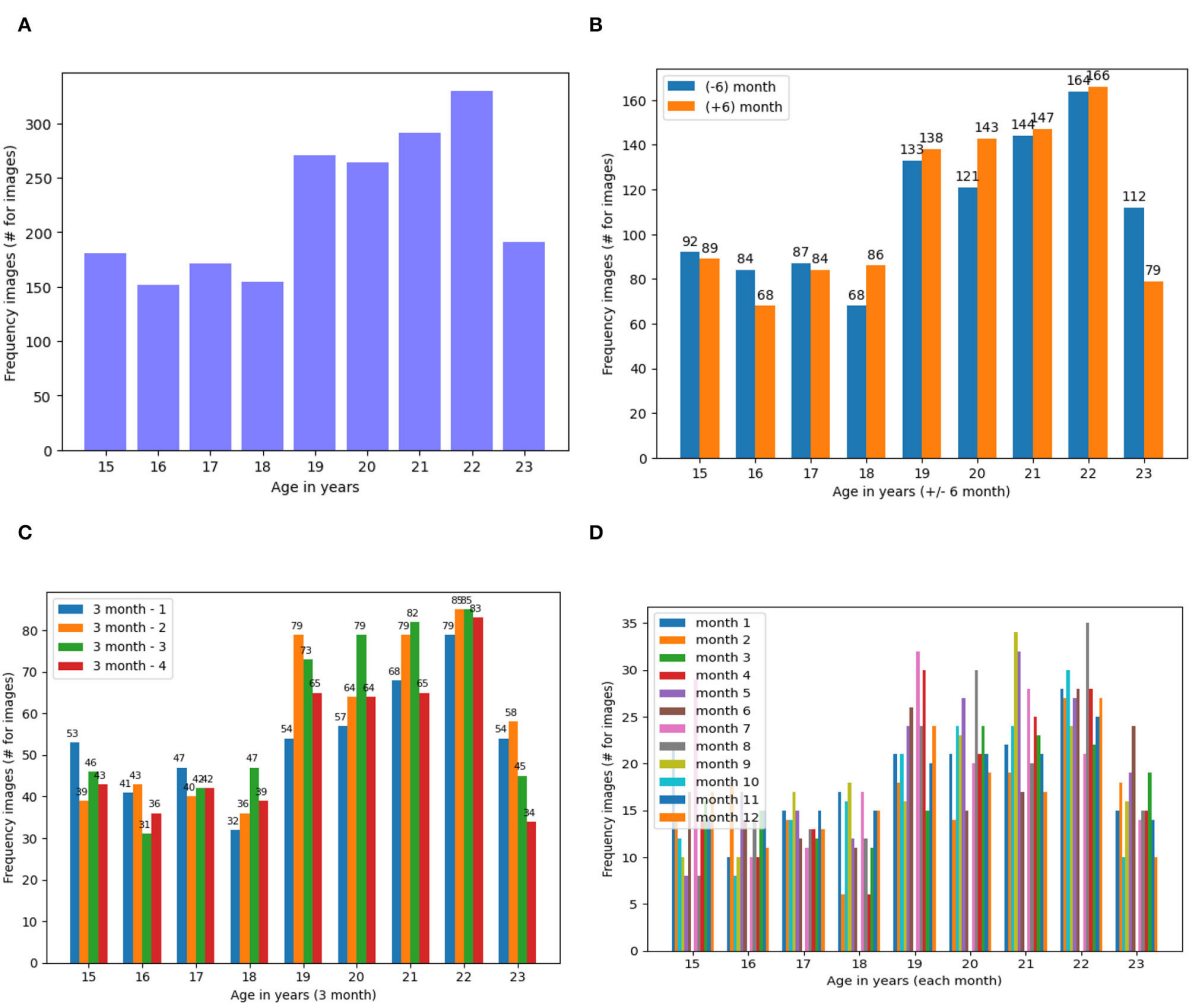
Frequency of Dataset Images Based on the Four States of the Dataset **(A)** Dataset on Year, **(B)**. On ± 6-Month, **(C)**. On ± 3 Month and **(D)**. On ± 1 Month. **(A)** Frequency of dental images for years dataset. **(B)** Frequency of dental images for ± 6 months dataset. **(C)** Frequency of dental images for ± 3 months dataset. **(D)** Frequency of dental images for ± 1 months dataset.

We elucidated the frequency of the dataset generated as the result of data augmentation when augmented more than 150, 68, 32 and 7 images in 1 year, 6 months, 3 months and 1-month categories, respectively (Figure 4).

### CNN Model Initial Accuracy on Four Different States of the Dataset

As the first experiment, we applied a CNN model on two other dataset states. In stage one, training and validation accuracy were evaluated from the original dataset without any pre-processing and data augmentation. In stage two, training and validation accuracy were assessed post-pre-processing operations on the data augmentation operations (Table 2).

**Table 2:** Experimental result on a dental dataset based on CNN.

		**Accuracy %**			
**Dataset**	**Train/Valid**	**Dataset on year**	**Dataset on ±6 month**	**Dataset on ±3 month**	**Dataset on ±1 month**
Original	Train Accuracy	77.85	76.24	72.41	70.26
	Valid Accuracy	58.91	43.72	38.75	23.29
Augmented- pre-processed	Train Accuracy	98.84	98.58	98.75	96.99
	Valid Accuracy	97.43	98.44	98.13	95.62

We observed that pre-processing and data augmentation operations consistently improved the accuracy by approximately 20% and 40% for training and validation, respectively (Table 2).

**Figure 5:**
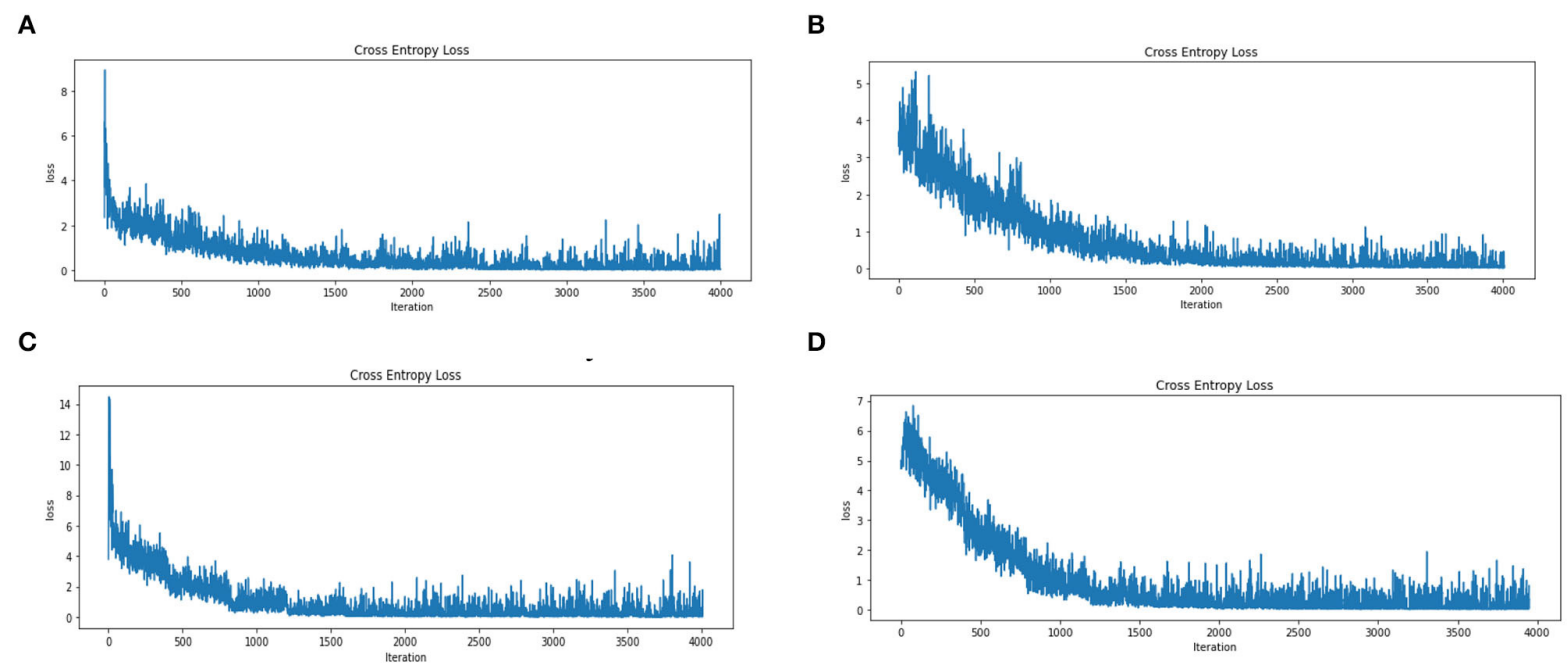
Error Loss for Training on A Dataset Based on CNN Model on the Four States of the Augmented Dataset **(A)**. Dataset on Year **(B)**. On ± 6-Month **(C)**. On ± 3 Month and **(D)**. On ± 1 Month. **(A)** Error loss for dataset years. **(B)** Error loss for the dataset with ± 6 months. **(C)** Error loss for the dataset with ± 3 months. **(D)** Error loss for the dataset with ± 1 months.

We then evaluated the loss reduction for each step (i.e., four separate modes) of this data set (1 Year, ± 6 months, ± 3 months, and ± 1 months) with 4,000 repetitions (i.e., epoch equivalent to 10 with about 400 batch size 16). Note that the results are obtained on cross-validation with 80% training and 20% validation and segmentation on dataset images. We observe a sharp decay in loss reduction values for both the 1 year and ± 3 months dataset, while the ± 6 months and ± 1 month dataset showed a more gradual decay (Figure 5).

**Figure 6:**
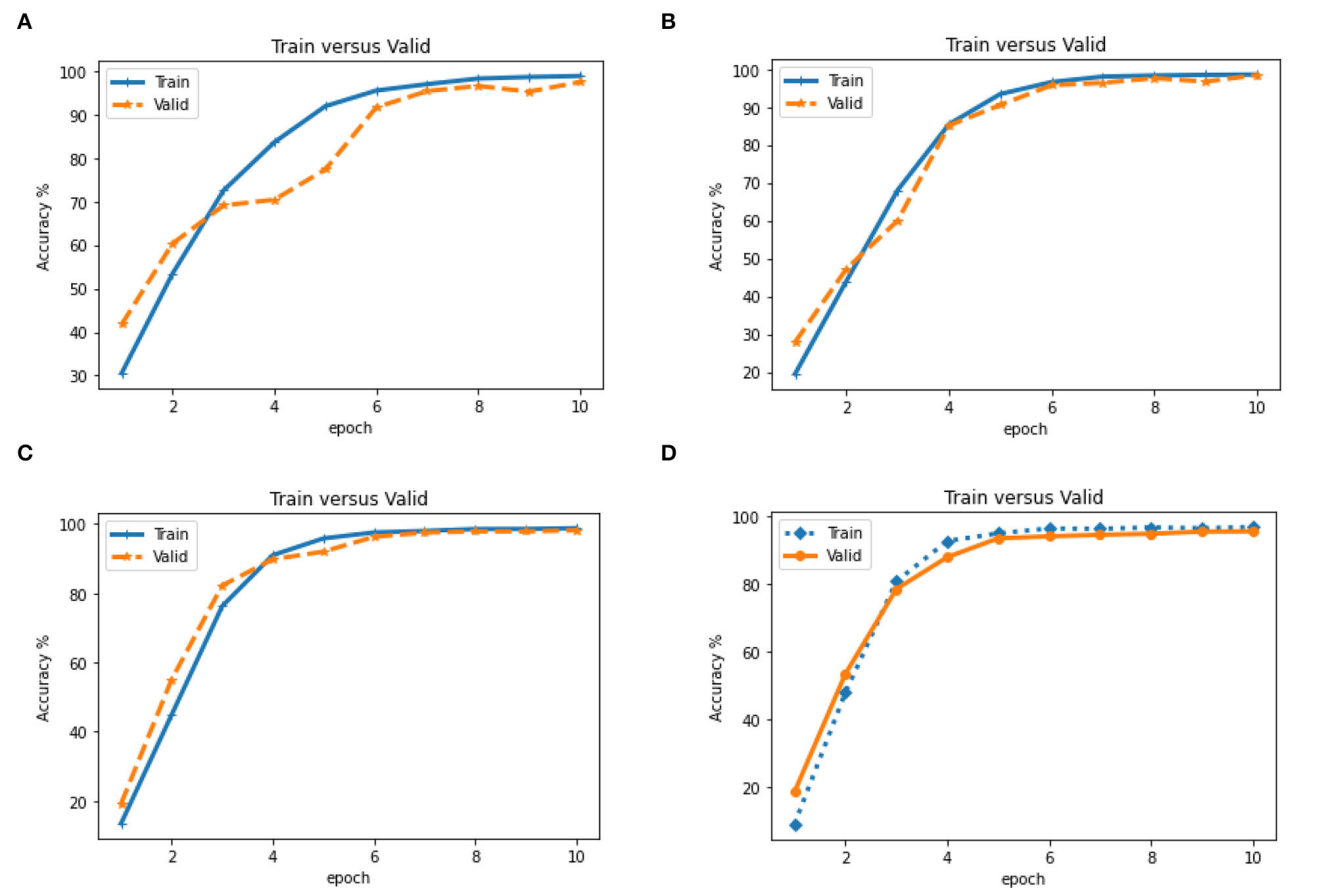
Evaluation of accuracy (Train vs. Valid) in different epoch based on CNN model on four states of the dataset **(A)**. Dataset on Year **(B)**. on ± 6-month **(C)**. on ±3 month and **(D)**. on ± 1 month. **(A)** Train vs. Valid for dataset years. **(B)** Train vs. Valid for the dataset with ± 6 months. **(C)** Train vs. Valid for the dataset with ± 3 months. **(D)** Train vs. Valid for the dataset with ±1 months.

We also evaluated the accuracy of the proposed model on train and validation data in 10 epochs. We observed that the accuracy of train data is generally slightly higher than validation data (Figure 6). Therefore, the upward trend inaccuracy at 10 epochs indicates an increase in CNN learning.

### Combining HCNN-KNN Enhanced the Accuracy of the Model

We used a combined methodology of convolutional neural networks and the nearest neighbor (classification) to further enhance the model's accuracy. The resulting hybrid model (HCNN-KNN) contained three main CNN, PCA, and KNN layers. Our findings showed that the KNN classification with 500 features selected from “Fully_PCA” (PCA data transform after fully connected in CNN) had increased the accuracy of the original data to approximately the same level of augmented data to 99.98, 99.96, 99.87, and 98.78 for 1 year, ± 6-months, ± 3-months, and on ± 1-month dataset, respectively. Intrinsically, we observed accuracy for train data to be equal to 100% (Table 3). This outcome indicated is fully trained using the training dataset. It is noteworthy that the results obtained for k-nearest neighbors value k = 1 in the proposed HCNN-KNN hybrid model. We have also evaluated the KNN method in the proposed HCNN-KNN hybrid model with a “k” value of 2 and 3 (Table 4). Model validation is essential for building a model and is susceptible to common pitfalls when splitting the training dataset (59, 60). To evaluate the possibility of overfitting pitfall resulting from splitting the training dataset, particularly for small ks (e.g., *k* = 1–3), we further validate the model using a dataset of dental images separate from the data used for initial training and validation; contained its special features. The unique features of these images are related to different races, different genders and are selected in the age range of 15 to 23. While obtaining acceptable results on these data indicates the proper performance of the KNN classifier even with k = 1.

**Table 3:** Experimental result on a dental dataset based on HCNN-KNN.

		**Accuracy %**			
**Dataset**	**Train/Valid**	**Dataset on Year**	**Dataset on ±6 month**	**Dataset on ±3 month**	**Dataset on ±1 month**
Original	Train Accuracy	100	100	100	100
	Valid Accuracy	99.93	99.84	99.74	98.15
Augmented	Train Accuracy	100	100	100	100
	Valid Accuracy	99.98	99.96	99.87	98.78

**Table 4:** Accuracy results of K = 1, 2, 3 on the validation data.

	**Accuracy %**			
**K**	**Dataset on Year**	**Dataset on ±6 month**	**Dataset on ±3 month**	**Dataset on ±1 month**
K = 1	99.98	99.96	99.87	98.78
K = 2	59.26	55.61	52.64	51.20
K = 3	43.89	41.64	38.02	33.77

### Evaluation of Different Cross-Validation

Different cross-validation of datasets was applied to evaluate our method (e.g., 50–50, 70–30 and 20–80 train-valid cross-validation, Table 5).

**Table 5:** Evaluation of accuracy on different cross-validation on a dataset.

			**Accuracy on different Cross-Validation (valid-Train)**		
**Dataset Type**	**Methods**	**Train/Valid**	**20-80**	**30-70**	**50-50**
Dataset on Year	CNN	**Train**	98.84	98.16	97.20
		**Valid**	97.43	94.08	85.58
	HCNN-KNN	**Train**	**100.00**	**100.00**	**100**
		**Valid**	**99.98**	**99.97**	**99.95**
	Resnet (Deep model)	**Train**	99.04	98.83	98.80
		**Valid**	98.25	95.75	86.03
Dataset on ± 6 month	CNN	**Train**	98.58	98.50	96.75
		**Valid**	98.44	94.72	86.66
	HCNN-KNN	**Train**	**100.00**	**100.00**	**100**
		**Valid**	**99.96**	**99.78**	**99.78**
	Resnet (Deep model)	**Train**	98.66	98.53	98.42
		**Valid**	98.69	95.47	87.43
Dataset on ± 3 month	CNN	**Train**	98.75	98.48	97.77
		**Valid**	98.13	96.25	84.61
	HCNN-KNN	**Train**	**100.00**	**100.00**	**100.00**
		**Valid**	**99.87**	**99.82**	**99.68**
	Resnet (Deep model)	**Train** **Valid**	98.69 98.44	98.74 96.42	98.70 86.23
Dataset on ± 1 month	CNN	**Train**	96.99	97.12	94.46
		**Valid**	95.62	92.40	80.32
	HCNN-KNN	**Train**	**100.00**	**100.00**	**100.00**
		**Valid**	**98.78**	**98.52**	**98.29**
	Resnet (Deep model)	Train	97.25	97.24	97.15
		Valid	95.63	93.16	90.27

*Bold value is the maximum accuracy compared to the others results*.

### Evaluation of Proposed Model on Second Dataset (Test Dataset)

**Figure 7:**
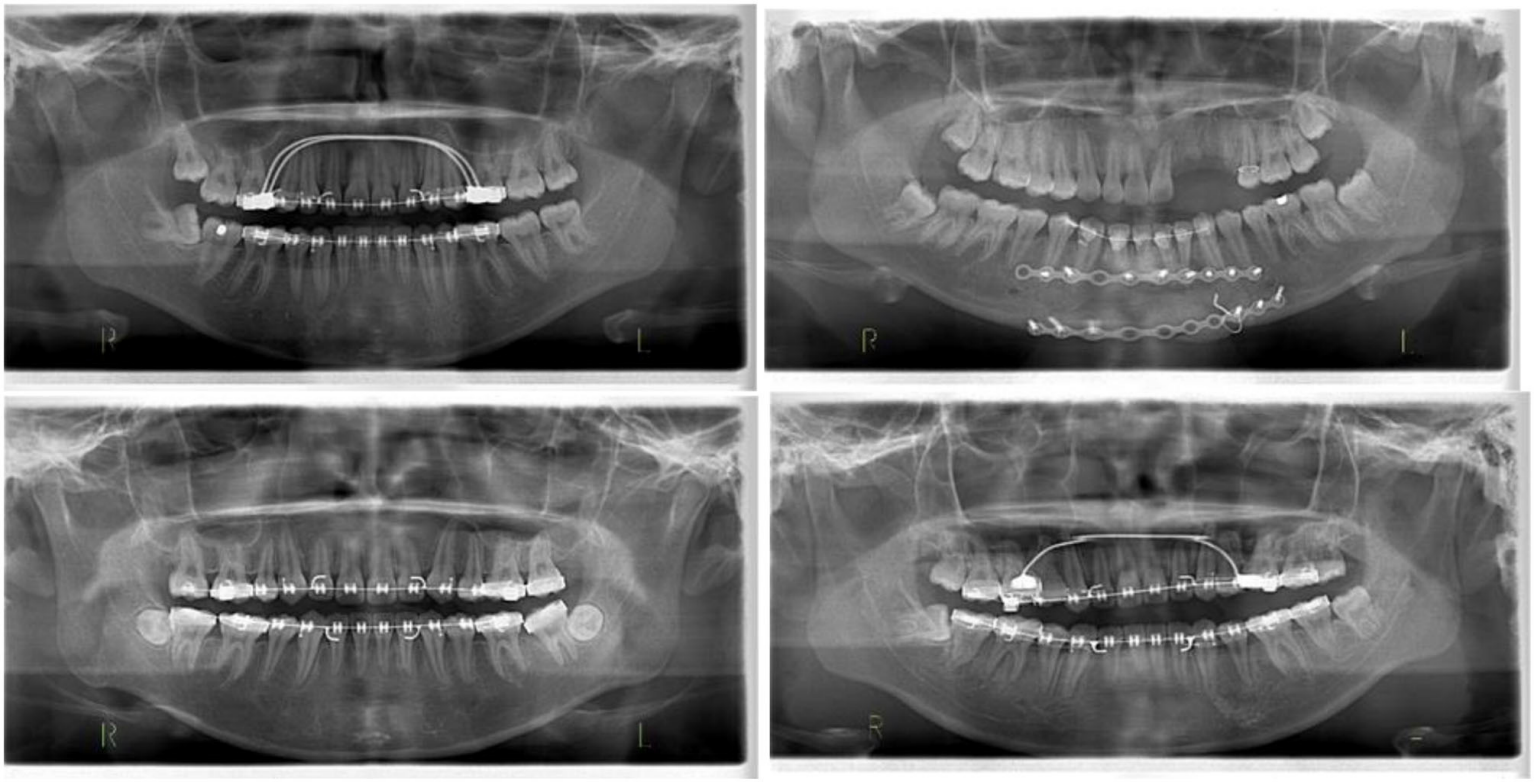
Different images of test data to evaluate the orthodontics (malignant) of the proposed model.

One of this study's innovative and key features is the additional validation step in which a set of images from a diverse cohort has been used to further validate the proposed model. After the initial training and validation, the images are only available to the model. The dataset had a combination of 130 X-Ray OPG images with different races (e.g., Malay, Indian and Chinese), with distinct features, various age ranges and normal distribution of ordinary dentistry, orthodontic and malignant images (Figure 7).

As Table 6 shows, the proposed HCNN-KNN model has obtained better results on the test data. Getting satisfactory results on a new dataset outside the dataset images indicates the performance of the proposed model. Thus, it seems that the proposed HCNN-KNN model has obtained superior results on the tested dataset. Obtaining satisfactory results on a new dataset outside the dataset images indicated the outstanding performance of the proposed model (Table 6). The HCNN-KNN model effects have also been tested for k 1, 2, and 3 (Table 6). The outcome shows the proper performance of the proposed model for k = 1. In KNN, where k is larger, the distance becomes more critical, overcoming the KNN principle that closer neighbors have the same density or classes. Since the classes in our dataset are very near to each other and are pre-processed with noise reduction, the result shows acceptable accuracy at K = 1.

**Table 6:** Accuracy Result of the Proposed HCNN-KNN Model on Test New Dataset.

			**CNN**	**HCNN-KNN**
All test Data	**Test data (130 dental images)**	22.93	**K** **=** **1 99.48**
			K = 2 74.61
			K = 3 70.22
Based on Different Races	**Malay** **(71 Images)**	25.23	**K** **=** **1 100**
			K = 2 76.05
			K = 3 73.23
	**Indian** **(16 Images)**	20.61	**K** **=** **1 93.75**
			K = 2 75.00
			K = 3 68.75
	**Chinese** **(43 Images)**	21.37	**K** **=** **1 97.67**
			K = 2 72.09
			K = 3 69.76

*Bold value is the maximum accuracy compared to the others results*.

**Figure 8:**
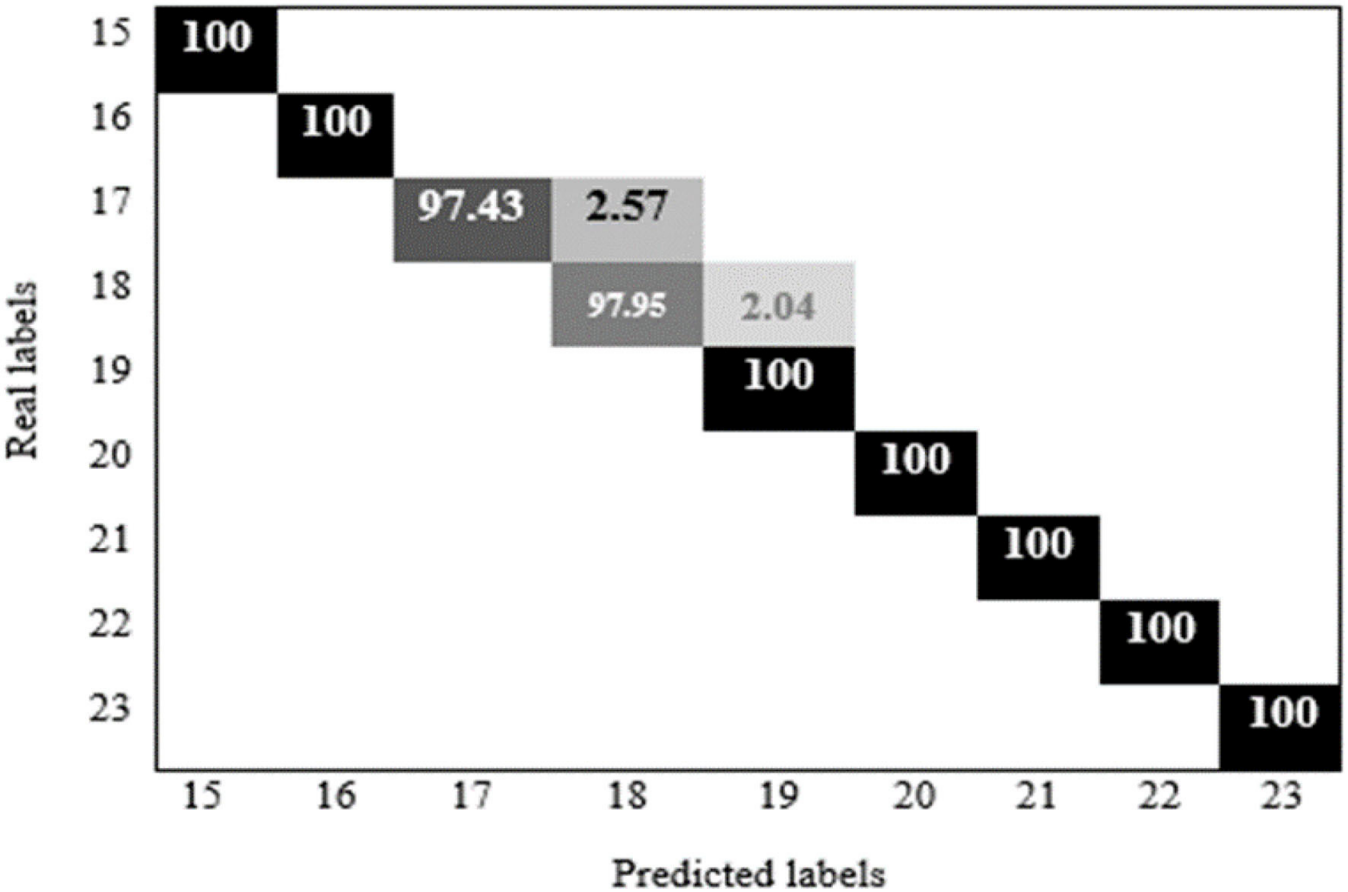
Confusion matrix of hybrid HCNN-KNN proposed model application on enhancement dataset.

Figure 8 shows the confusion matrix of 97.95 % for the 18-year-old age range which was correctly estimated, and 2.04 % was incorrectly identified for the 19-year-old.

### Models Accuracy

The collected data and the proposed model have been tested with other models such as ResNet, CNN, GoogLeNet Inception (Table 7). Our finding shows that the hybrid HCNN-KNN model has obtained much higher accuracy than different conventional classification algorithms tested.

**Table 7:** Comparison between the proposed HCNN-KNN model and other studies in bam based on dental images.

**References**	**Accuracy**	**Models**
**CNN**	**97.43**	**Other model test result**
**HCNN-KNN**	**99.98**	**The proposed hybrid model result**
**ResNet**	**98.25**	**Other model test result**
**GoogLeNet Inception**	**55.69**	**Other model test result**
SVM	92.1	(11)
FNN-TLBO	89.00	(61)
Multilayer Perceptron	90.00	(54)

*Bold value is the maximum accuracy compared to the others results*.

The novelty of our study is that to the best of our knowledge, no other study reported measurements of ± 6 months, ± 3 months, and ± 1 month so far, but only compared by the year. However, the results of this proposed model are compared with previous studies by years of accuracy. For example, Avuçlu used the Multilayer Perceptron model with 1,315 dental OPG images, and the highest accuracy was 90% just for accuracy of the years. Also, in 2020, Hussein used the SVM algorithm for a population of 1,429 dental radiographs, and they only achieved 92% accuracy for years. Therefore, based on Table 7, the results of the comparative studies for accuracy by years are still below the accuracy achieved in this study which is 99.98.

## Conclusions

For the first time, with precision to the range of +/- 6 months, this novel implementation developed an HCNN-KNN model, used for BAM to increase the existing model's accuracy and prevent CNN overload situations. We considered more specific cases of bone age measurement with the help of Dental X-Ray OPG images to solve the problem of bone age measurement in determining the age range of 15 to 23 years based on the Year, ± 6 months, ± 3 months, and ± 1 month. We integrated the methodology of convolutional neural networks for extraction and analysis of information and features in dental images) and the nearest neighbor (classification method). The primary purpose of this proposed model is to use KNN instead of SoftMax in the fully connected layer to increase the performance of convolutional networks in the classification phase. Using principal component analysis as data transform and feature dimensionality reduction in a fully connected layer before classifying KNN as Fully_PCA. The primary purpose of this method is to transfer data to space with more specific information to increase the performance of the classification phase.

Our proposed model achieved the accuracy of 99.98, 99.96, 99.87, and 98.78 in 1 year, ± 6-month, ± 3-month, and ± 1-month range, respectively. Our proposed method evaluated different cross-validation of the dataset. 50–50 train-valid, 70–30 train-valid and 20–80 train-valid cross-validation on the dataset. Evaluating the proposed model on a new dataset with different races also proved the superior performance of the model. The benchmarking with current existing models also showed that the HCNN-KNN model is the best model for bone age measurement.

## Data Availability Statement

The raw data supporting the conclusions of this article will be made available by the authors, without undue reservation.

## Author Contributions

FS and SB designed the study. FS wrote the manuscript, collected the data, carried out the analyses, including statistical analyses and the implementation of machine learning methods and generated all figures and tables. FS, SB, PO, and HA-R edited the manuscript. HA-R and PO were not involved in any analysis. All authors have read and approved the final version of the manuscript.

## Conflict of Interest

The authors declare that the research was conducted in the absence of any commercial or financial relationships that could be construed as a potential conflict of interest.

## Publisher's Note

All claims expressed in this article are solely those of the authors and do not necessarily represent those of their affiliated organizations, or those of the publisher, the editors and the reviewers. Any product that may be evaluated in this article, or claim that may be made by its manufacturer, is not guaranteed or endorsed by the publisher.
